# Pneumonia

**Published:** 1884-09

**Authors:** 


### PNEUMONIA.

INFLAMMATION OF THE LUNGS is a disease that attacks suddenly; is always alarming, and, unless promptly and judiciously treated, is very fatal, particularly to persons over fifty years of age. Young persons usually recover from the inflammatory stage; but very many die, perhaps years afterward of lung disease, of which this was the commencement; hence the importance of knowing *the conditions* that are liable to produce the disease; *the symptoms* that denote its existence, and *the best means of arresting* a malady whose march is always rapid and destructive.

As to *the conditions that are liable to produce the disease:* I have observed that a person who remains in a warm, but dry and well ventilated room, long enough for the skin to become moist with perspiration, and then goes out of doors where the temperature is considerably lower, is liable to have an attack of *influenza* more or less severe, *but the lungs usually remain intact.* If, however, the the room is *ill ventilated,* and many people are congregated together, as at church, a concert, or other assembly, the air becomes poisoned by exhalations from the lungs and bodies of those present, and, being re-breathed, acts as an irritant to the delicate tissues of the lungs, lessening their power of resistance to disease; under these circumstances, sudden exposure to cold is almost certain to be followed by *inflammation of the lungs,* of greater or less severity, depending on the age and vigor of the person attacked.

As these are facts of vital importance to all, and a general knowledge of them would save thousands of valuable lives every year, they should not be forgotten. I do not remember to have seen them stated by any other medical writer, though they could hardly have escaped the notice of all experienced physicians. It is perhaps within bounds to say that nine-tenths of the premature deaths from diseases in this country are due to blood poisoning, caused by inhaling noxious gases in place of pure air. This, howver, I will make the subject of another article.

PNEUMONIA is an inflammation of the spongy tissue of the lungs. There are numerous varieties of the disease, depending upon its location and extent; but this is of but little importance to the general reader. There are also various symptoms depending on the severity and the location of the disease, or its complication with other maladies. It would require too much space tc refer to these in detail, and I can only give such as are necessary to determine the nature of the complaint. The first symptom usually observed is a chill, soon followed by fever, labored breathing, severe pain in some portion of the chest—but more frequently about one inch below the nipple, over the lung most affected. This is the first or

*congestive stage* of the disease; and, if the treatment is prompt and judicious, it ordinarily extends no farther; but when it enters the next or *inflammatory stage*, it becomes formidable under all circumstances, and terribly relentless to those of advanced years, because the recuperative powers of the system are feeble.

SYMPTOMS: The signs that usually characterize the *congestive stage* of the disease, occasionally seem to be wanting or are not noticed, and the patient finds that he has, almost unconsciously, reached the *inflammatory stage*. But, although not observed, the disease has really passed through the first or *congestive stage*, though there may have been simply a feeling of weariness or an aching of the muscles to denote it. The *second stage* is characterized by increased fever, more rapid breathing, and more or less cough, followed by a little mucous expectoration, which soon increases in quantity, and is stained with blood, so as to have a reddish appearance. The tenacity of the sputa is so great that it adheres to the vessel when turned bottom upward. This rusty-colored expectoration is the most characteristic sign of pneumonia. The disease seldom attacks both lungs at the same time, and it more frequently occurs in the right lung.

IN THE THIRD OR SUPPURATIVE STAGE, portions of the lungs becomes *hepatized*, that is, like the liver, in density. These hepatized portions ulcerate and form cavities. This condition is extremely dangerous, particularly to patients who inherit a tendency to consumption. I have thus given an outline of the various stages of this dreaded disease. It is sufficient, I believe, to put the reader on his guard against it; or, if not, he will be able to recognize the enemy and expel it before a foot-hold is gained.

REMEMBER that *Pneumonia* may be almost always avoided by a proper observance of the laws of health, and that, in its *first stage*, it is almost always curable.

We have not space to give the most approved treatment of the disease in its various stages, but will do so in a subsequent number of the JOURNAL OF HEALTH.

